# Differentiating Normal Healing From Infection in Total Joint Arthroplasty: An Educational and Diagnostic Tool for Emergency Providers Evaluating the Postoperative Patient

**DOI:** 10.1111/acem.70414

**Published:** 2026-09-27

**Authors:** Thomas W. Brown, Andrew T. Carusi, Michael Langworthy

**Affiliations:** ^1^ Tufts University School of Medicine Boston Massachusetts USA; ^2^ Southcoast Health New Bedford Massachusetts USA; ^3^ Department of Orthopedic Surgery Southcoast Health New Bedford Massachusetts USA

**Keywords:** diagnostic algorithm, emergency department, periprosthetic joint infection, postoperative care, surgical site infection, total joint arthroplasty

## Abstract

Patients frequently present to the emergency department (ED) with pain, swelling, or erythema in the early period following total joint arthroplasty (TJA). Differentiating normal postoperative inflammation from superficial surgical site infection (S‐SSI) or early periprosthetic joint infection (PJI) can be difficult due to overlapping clinical and laboratory features, particularly within the first 30 days after surgery. We seek to provide emergency providers with an efficient and evidence‐based framework for differentiating normal healing from early infection following TJA. We accomplish this by reviewing postoperative physiology, temporal response of inflammatory markers and synovial fluid analysis, and existing diagnostic criteria for infection. Normal healing after TJA produces predictable inflammatory responses that commonly exceed thresholds established for chronic PJI diagnosis. Therefore, early PJI diagnosis should utilize a time‐dependent interpretation of synovial fluid, while S‐SSIs remain primarily clinical diagnoses. A structured diagnostic algorithm can safely reduce unnecessary testing and costs, protect against medicolegal pressures, and provide evidence‐based practices for effectively detecting early infection following TJA. This is crucial in the setting of the new Transforming Episode Accountability Model (TEAM) that bundles TJA payment.

## 1 Introduction

Early periprosthetic joint infections (PJIs) and superficial surgical site infections (S‐SSIs) can be difficult to differentiate from normal physiologic healing following total joint arthroplasty (TJA). Early postoperative infection, defined in this paper as site infection within 30 days of surgery, includes both PJIs and S‐SSIs as serious complications of TJA [1]. S‐SSIs can progress to PJIs, which in turn can lead to artificial joint and tissue destruction, increased morbidity, decreased quality of life, and heightened healthcare costs [2, 3]. The overlap of PJIs and S‐SSIs with normal healing is particularly important in acute care settings, as the emergency department (ED) is often the first point of contact for patients presenting with suspected postoperative infection. Patients with true infection can have variable presentations, ranging from acute pain to sepsis [4]. Patients may have signs and symptoms that can also be mistaken for noninfectious causes, such as hemarthroses or crystallopathies [5, 6]. Additionally, early postoperative CRP and ESR are elevated, peaking within days and taking weeks to months to return to normal levels [7, 8, 9]. Because of these natural elevations in inflammatory markers, it can be difficult to distinguish normal healing from true infection. These factors increase uncertainty, which may cause providers to defensively over‐test to circumvent misdiagnosis.

This uncertainty is especially important in the context of shorter postsurgical stays, more effective intra‐operative pain management, and a new payment method for TJA. As more advances are made in surgical techniques and postoperative pain management, postoperative hospitalization stays will continue to decrease. Since the early 2000s, length of stay following TJA has decreased from an average of 4 days to 1–3 days [10, 11, 12]. As orthopedists generally do not see their patients again until roughly 10–14 days post TJA, the “black‐hole period” where patients are not seeing a provider after initial discharge will continue to increase [13]. Although shorter length of stays following TJA do not increase total ED visits, patients with shorter stays are more likely to present to the ED with concerns of pain and/or swelling than patients with longer hospitalizations following TJA [14, 15]. Furthermore, current intra‐operative pain management technologies can effectively control pain for up to 3–4 days post‐surgery, compared to 12 h of analgesia with previous modalities [16, 17, 18]. This means patients will experience a significant expected increase in pain around day 4, which exists in the “black‐hole period.” Another increase in pain occurs with peak swelling, roughly 6–8 days following surgery [19]. These factors combined may lead to more patients visiting the ED several days following TJA. To complicate matters further, reimbursement for care related to TJA in the 30 days following surgery will now be bundled into the total cost of TJA as of January 1, 2026. Hospital systems will receive no additional reimbursement for TJA‐related ED visits or diagnostic work‐ups within the initial 30‐day period [20]. This further necessitates an enhanced knowledge of the early postoperative period and a streamlined diagnostic algorithm to rule out infection from normal healing.

## 2 Familiarizing Emergency Providers With Enhanced Recovery After Surgery (ERAS) Protocol

The Enhanced Recovery After Surgery (ERAS) Protocol is a heterogeneous collection of interventions that surgeons utilize to reduce hospital stays and postsurgical complications [21]. One aspect of ERAS includes local anesthesia. New extended‐release formulations of bupivacaine, such as liposomal bupivacaine or bupivacaine/meloxicam, can effectively control pain for roughly 72–96 h post‐surgery and have been shown to reduce hospital stays, improve pain, and decrease opioid use [16, 18, 22, 23]. Roughly 8% and 10% of patients with recent TJA present at least once to the ED within 30 and 90 days of surgery, respectively, with pain and swelling being the most frequent reasons for ED visits [24, 25]. Naturally, pain is anticipated to increase once patients begin physical exercise and rehabilitation [26]. However, a decreased perception of pain in the first few days post‐surgery may unintentionally provide patients with overconfidence and enable them to prematurely overexert themselves in this period. Overexertion may lead to heightened inflammatory response that interferes with normal inflammation‐mediated healing, increasing pain and prolonging recovery [27]. Furthermore, once the effects of extended‐release formulations wear off, patients can experience a sudden increase in pain levels. This phenomenon is known as rebound pain [28, 29]. Furthermore, pain may radiate into peripheral tissue as a means of secondary sensitization to pain following joint manipulation during arthroplasty [30]. Rebound and radiating pain, especially when coupled with recent overexertion, may concern patients enough to initiate ED visits.

Another area of ERAS protocol that emergency providers should be familiar with is orthopedic wound dressings. Orthopedists commonly utilize hydrofiber wound dressings, such as Aquacel, for TJA. Hydrofiber wound dressings are designed specifically to wick away exudative drainage and can give the appearance of significant bleeding though no clinically worrisome bleeding may be present. This is normal and should not be misidentified as significant blood loss or prevent the emergency provider from adequately investigating the surgical site (Figure 1).

**FIGURE 1:**
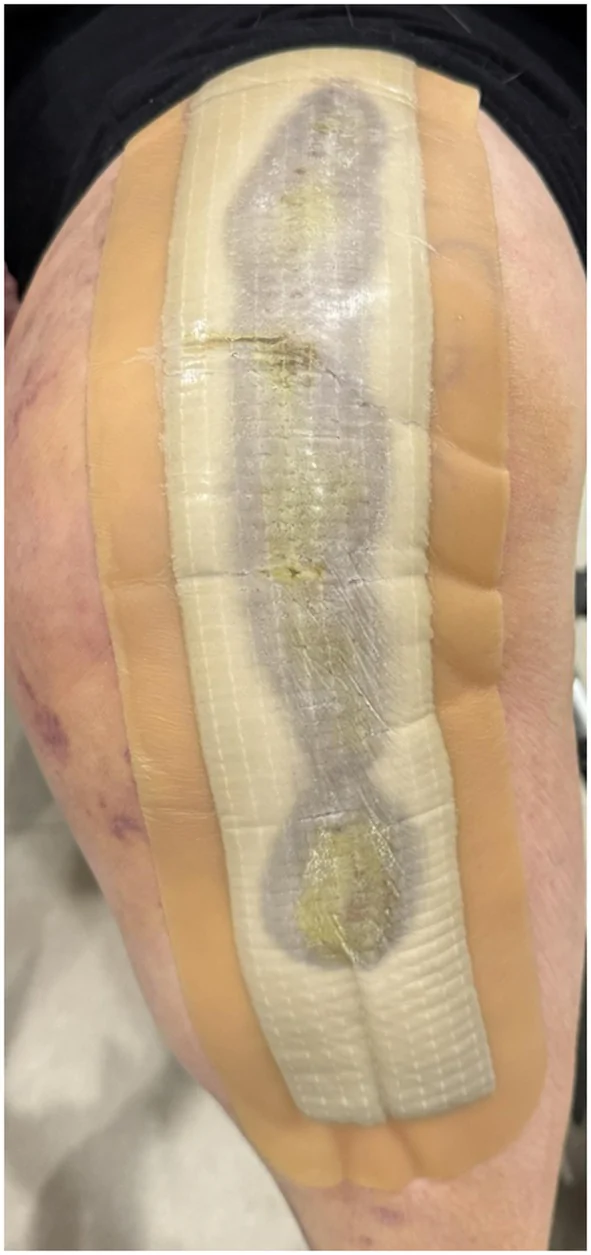
Image of an intact Aquacel dressing appropriately covering a left total knee arthroplasty. As intended, the hydrofiber material wicks away blood and serosanguinous drainage from the incision site. Providers who are unfamiliar with this dressing may confuse this phenomenon for active bleeding. The patient in this image consented and all identifiers have been removed.

## 3 Differentiating Normal Healing From Superficial Infection or Septic Joint

TJAs are a significant injury to the body and can be an expected source of acute systemic inflammation [31]. In normal healing of TJAs, providers should expect to find mild, localized erythema, swelling, and warmth at the incision site that should progressively resolve. There should be no purulent drainage, though serous drainage is not uncommon [32, 33]. The timing of resolution of these clinical signs will vary between patient cases. No studies specifically address how long localized erythema can last, though it is generally reported to last several weeks, with some patients experiencing lingering erythema for 3–6 months. Swelling increases by roughly 10% each day for the first few days post‐TJA, peaking between day 6 and day 8 before slowly decreasing [19]. The amount of edema varies greatly between patients [34]. In general, patients can expect to still have swelling roughly 10% above baseline at 90 days post TKA operation [35]. Warmth is a natural part of inflammation and healing. One meta‐analysis found that knee joint temperature following TJA can remain elevated at 12 months post‐surgery [36]. In normal healing, there should be no wound dehiscence. Besides the exceptions mentioned in above sections, pain generally decreases progressively. Though, between 10% and 30% of patients with TJA report on‐going pain [37].

Importantly, benign rashes may rarely appear. First, flat, erythematous changes to skin near the surgical incision may occur during the first week; these changes should not be associated with swelling or wound drainage. The definite cause is unknown, but may be due to tape sensitivity or other tissue insult. Most of these skin findings will self‐resolve [38]. Second, metal hypersensitivity can cause an erythematous, scaly, and pruritic rash between 2 months and 2 years following TJA. The rash may also produce exudate and can be local to the surgical site or more widespread [39]. Third, TKA patients may rarely experience a SKINTED rash (Surgery of the Knee, INjury to the infrapatellar branch of the saphenous nerve, Traumatic Eczematous Dermatitis); this is an autonomic denervation dermatitis that can produce a scaly, erythematous rash associated with numbness [40, 41]. Fourth, reticular telangiectatic erythema (RTE) may occur days to years after surgery. RET is a blotchy and blanching erythema near the surgical site. The cause is unknown, but it is thought to be due to obstruction of blood flow or a very low‐grade infection [42, 43].

Although patients generally have no systemic symptoms of illness after TJA, the normal inflammatory cascade can cause mild fever [44]. Fever can occur in the first 48 h and up to 4 days following TJA. Fevers within the first 48 h are generally non‐infectious, though *Strep. pyogenes* and *Clostridium* species can cause early infectious fevers. Fevers occurring after 4 days are more suggestive of infection [38].

Emergency physicians should expect to find increased inflammatory markers in normal healing following TJA. Early postoperative inflammatory markers are elevated in response to normal surgical healing. CRP peaks 2–3 days post‐operatively and takes three or more weeks to return to baseline [45, 46, 47, 48]. ESR peaks at day five and takes months to decrease to baseline [49]. Mean ESR levels within 6 weeks of TJA can range from 46 to 75 mm/h in non‐infected patients with a median value of 26 mm/h. Mean CRP levels within the same time frame can range from 30 to 88 mg/L with a median value of 12 mg/L [50, 51, 52].

The normal inflammatory cascade from TJAs has been shown to double the release of WBCs from lymphatics [44]. One study found that on average, serum WBC in TJA increased by 3 × 10^6^ cells/μL by day 2 post‐operation, before decreasing to slightly higher than the preoperative baseline at day 4. Mean maximum serum WBC in noninfected patients within 3 weeks of TJA was found to be 10.7 × 10^6^ cells/μL [53].

Importantly, synovial WBC level will also be elevated following in normally‐healing TJAs after surgery in response to transient inflammatory changes. In the first 45 days following TJA, normal synovial WBC count ranges between 2500 and 4200 cells/μL with a PMN percentage of roughly 63%–77% [50, 52, 54]. This differs from normal WBC count in the native, noninfected joint, where WBC count is generally considered to be less than 200 cells/μL. For context, synovial WBC counts in native joints are typically < 2000 cells/μL in noninflammatory conditions (e.g., osteoarthritis), 2000–50,000 cells/μL in inflammatory diseases (e.g., rheumatoid arthritis), and > 50,000 cells/μL in infections [55].

Superficial surgical site infections (S‐SSIs) are infections of the subcutaneous space, which exists between the skin and muscle fascia [38]. S‐SSIs following TJA can cause cellulitis‐like and erysipelas‐like infections. Overall, S‐SSIs account for roughly 60% of surgical site infections in TJA [56]. Emergency providers can expect to find increasing erythema, warmth, and tenderness of the skin and subcutaneous tissue near the site of surgery. Superficial purulent drainage can be a diagnostic finding for S‐SSI, but may or may not be present at the site of incision [1, 38]. Studies in other surgical fields have found that site purulence is present in 26.7%–48.4% of S‐SSIs [57, 58]. Patients with S‐SSIs following TJA typically have no systemic symptoms. Fever, leukocytosis, and malaise may occur in S‐SSIs, but these signs and symptoms are more associated with deeper infections [1]. Importantly, there should be an absence of deep joint pain or increased joint effusion, as these findings would suggest a deep incisional infection (e.g., fascia or muscle infection) or organ/joint space infection (i.e., PJI). Furthermore, joint aspiration should be unrevealing as there is no involvement of deeper structures in S‐SSI [59]. S‐SSIs are also a risk factor for developing PJI, as superficial infections can progress to joint space infections [3]. S‐SSIs and cellulitis near the surgical site can often be conflated with one another. Cellulitis is an infection of dermis/subcutaneous tissue. Unlike S‐SSIs, which are generally localized and centered on the surgical site, cellulitis is diffuse with a center outside the surgical site. Peri‐incisional cellulitis may be categorized as a subsection of S‐SSI if it meets S‐SSI criteria listed in the next section, however, these terms are not generally synonymous [60, 61]. The combined incidence of S‐SSIs and cellulitis is roughly 0.47% in the early period following TJA [62].

Periprosthetic joint infections within 30 days of TJA fall into the category of early PJI, or PJIs that occur within 3 months of joint replacement. Early PJIs are usually attributed to direct inoculation at the time of surgery and can occur in 0.3%–1% of patients [2, 63, 64, 65]. Early PJIs are typically caused by virulent organisms, such as *Staphylococci*, gram‐negative bacteria, and *Enterococci* [65, 66]. Emergency providers can generally find increasing erythema and warmth at the surgical site, with the addition of increasing deep joint tenderness, stiffness, and effusion in patients with PJI [59]. Although the literature does not specifically quantify the incidence of symptomatology in PJI within 30 days of TJA, it is generally accepted that joint pain is the most common symptom in early PJI [59]. One study that excluded early presentations and diagnosed PJIs during joint revision surgery found that pain was nearly universal, present in roughly 96% of patients. Reduced range of motion was present in 84% of cases, effusion in 79%, erythema in 44%, and fever in 40% [67]. However, another study, where 90% of patients presented with early PJI, found purulent discharge was reported in 72% of patients, joint pain in 42%, erythema in 42%, and fever in 38% of patients [68]. This finding may suggest that joint pain may be less common in early PJIs, but as before, no study has quantified the incidence of symptoms within 30 days of TJA. Another study found wound drainage > 3 days post‐operation was associated with roughly 91% specificity for PJI, but only had a sensitivity of roughly 63% [69]. As mentioned, postoperative fever is nonspecific and generally only important in the correct clinical picture. For example, one study found that every patient who developed fevers within 30 days of TJA but lacked local signs of infection did not have PJI [70].

As PJIs progress, patients may develop sinus tracts with purulent drainage and extension of infection to superficial tissue [66]. Sinus tracts are pathognomonic for PJI, boosting a specificity for PJI between 97% and 100%, but are only present in a minority of patients with PJI [71]. Overall, sinus tract formation occurs in roughly 15% or less of PJIs [72, 73, 74]. Generally, sinus tracts tend to be more associated with delayed (3 to 12–24 months post‐TJA) or late (12–24+ months post‐TJA) infections [2]. Sinus tracts are uncommon in early PJI, occurring in only 5% of a cohort in which 90% of patients presented within 3 months of TJA [68].

Multiple studies have examined laboratory findings in early PJI. In patients with PJIs within 6 weeks of TJA, mean CRP levels were reported to be 171–192 mg/L and median CRP values were found to be 101–123 mg/L [50, 51, 52, 75]. Optimal CRP cutoffs for distinguishing infected versus noninfected TJA within 6 weeks were reported to range between 82 and 100 mg/L [50, 51, 52, 75]. In the same time frame, patients with PJI were found to have mean synovial WBC values between 50,825 and 92,600 cells/μL and mean PMN percentages between 89.6% and 93.3%; median WBC values were between 13,949 and 58,295 cells with median PMN percentages at 91% [50, 51, 52, 75, 76]. A systematic review and meta‐analysis found optimal cutoffs for synovial WBC count and PMN percentage to be 13,600 cells/μL and 90.5% at 3 weeks and 9338 cells/μL and 88% at 6 weeks from TJA [77]. The most refined temporal analysis of early PJIs is found in a study that evaluated synovial WBC and differential at the intervals of 0–15 days and 15–45 days following TJA. The optimal cut‐offs in differentiating PJI at 0–15 days were 21,003 WBCs/μL and 92% PMN. The optimal diagnostic threshold at 15–45 days was a WBC count of 5111 cells/μL and PMN% of 80% [78]. For completeness, mean ESR values in PJI patients within 6 weeks of TJA were found to be 69–80 mm/h [50, 51, 52]. However, because ESR remains elevated for a much longer duration compared to other measures, it is generally less sensitive, less specific, and less clinically useful than CRP, WBC, or differential for diagnosing early PJI [50, 52, 79]. In fact, one study evaluating 11,964 primary TKAs found no significant difference between ESR levels in infected versus noninfected joints investigated for possible PJI within 6 weeks of TJA [52]. Accordingly, ESR is not recommended for early PJI evaluation [80]. Serum WBC count is also generally not useful soon after TJA unless sepsis is suspected, as mean maximum serum WBC count in PJI patients within 3 weeks of TJA was found to be similar to noninfected patients (11.4 × 10^6^ cells/μL versus 10.7 × 10^6^ cells/μL, respectively) [53]. However, one study found that multiplying pre‐operative leukocyte count by the leukocyte count on day 2 post‐operation could produce a specificity for PJI at roughly 96% if the product was > 100 [69]. This equation may be useful in a limited window, though further research is needed. Overall, CRP and synovial WBC with differential are most useful in evaluating for possible PJI in the early setting.

It is important to note that normal healing can resemble signs and symptoms of sepsis. EM providers often rely on these signs when making clinical judgments, whether following SIRS criteria or newer protocols. Notably, only 40% of patients with true PJIs met Sepsis‐1 criteria (SIRS positive plus suspected infection) and only 44% of patients with PJIs were either SIRS positive or bacteremic [81, 82]. Therefore, SIRS criteria alone are not sufficient in evaluating the emergency patient with concerns involving early joint replacement. Newer sepsis criteria seek to improve upon the limitations of SIRS by emphasizing infection source with signs of organ dysfunction to diagnose sepsis. Further research is needed to evaluate how these changes impact evaluation of early TJA concerns in the emergency setting (Figure 2).

**FIGURE 2:**
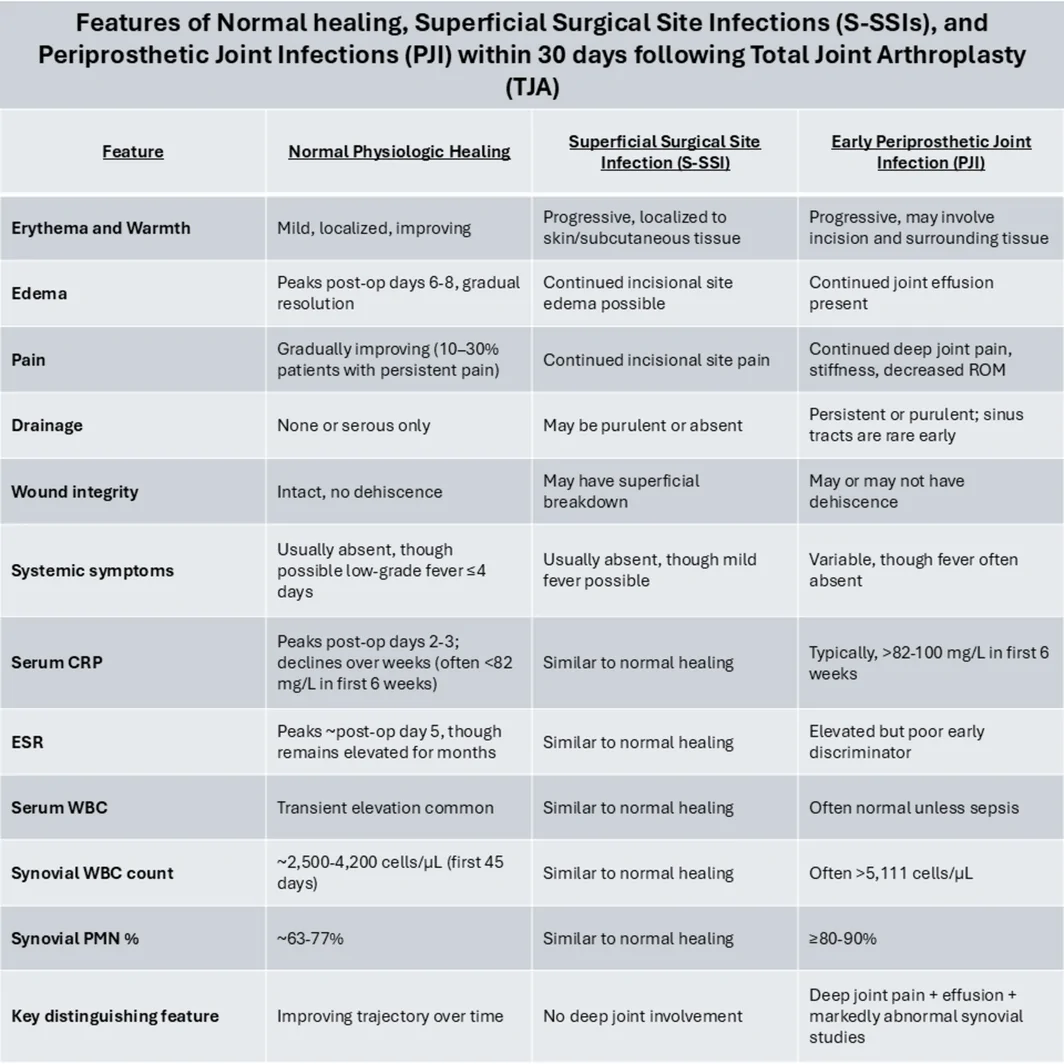
Representative features of normal healing, S‐SSIs, and PJIs within 30 days of TJA.

## 4 Diagnostic Algorithm

Emergency providers should obtain a detailed history, including the nature and timing of their recent TJA, their postsurgical course, and how their symptoms have evolved over time, including changes in ability to bear weight (Figure 3). Physical exam should include full inspection of the joint and surrounding tissues with complete removal of the patient's dressing. In addition to usual palpation, range of motion testing, neurovascular testing, and stability testing, providers should also examine adjacent joints and spine to evaluate for referred pain [83].

**FIGURE 3:**
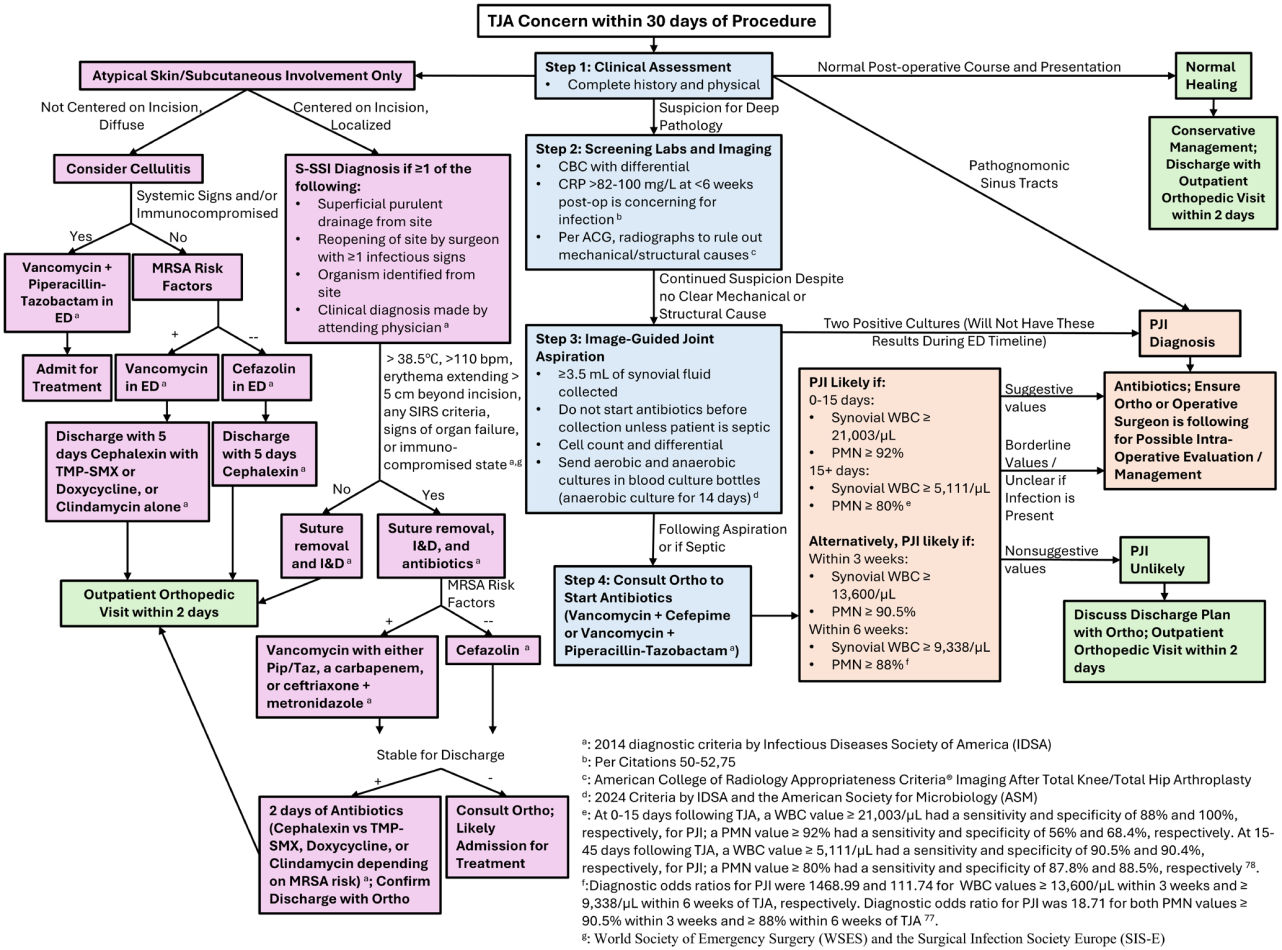
Diagnostic algorithm for TJA concern within 30 days following TJA.

The American College of Radiologists (ACR) recommends radiographs as “usually appropriate” in symptomatic patients following TJA if there are specific clinical indications warranting imaging evaluation, such as suspicion for mechanical or structural etiologies [37, 84]. However, if history and physical examination are consistent with only expected findings of normal healing as described in above sections, then emergency providers can safely defer imaging. Radiographs have a low sensitivity and low specificity for infection and are only utilized to evaluate for non‐infectious causes, such as periprosthetic fractures, component malposition, or early mechanical loosening [37].

If history or physical exam is not suggestive of normal postoperative healing, then further evaluation is required. Should superficial pathology be suspected, emergency providers may consider a diagnosis of S‐SSI. Importantly, S‐SSI is predominantly a clinical diagnosis, and diagnostic criteria are intentionally broad [61]. Following the Infectious Diseases Society of America guidelines, S‐SSIs following TJA can be diagnosed by meeting one of the four following criteria within 30 days of surgery:

1. Presence of purulent discharge from the surgical site.
2. Identification of an organism from the surgical site.
3. Clinical diagnosis of a surgical site infection by the surgeon or attending physician.
4. Deliberate reopening of the wound by the surgeon, accompanied by at least one associated infectious symptom, such as swelling, erythema, or localized pain or warmth (except if collected cultures return negative) [38]

In contrast to S‐SSI, cellulitis would appear more diffuse and is generally not centered on the incision site. A patient's cellulitis would only be considered a S‐SSI if it met the above criteria [60, 61].

If deeper pathology is suspected, then additional work‐up is needed. Although the American Academy of Orthopedic Surgeons (AAOS) recommends ESR, CRP, and serum interleukin‐6 testing in patients being evaluated for PJI, the AAOS also acknowledges that the interpretability of these tests can be difficult to assess in the early postoperative period [85]. Temporal consideration of labs in suspected early PJI must be taken. Serum CRP should be ordered and evaluated with higher evidence‐based thresholds in the early period compared to chronic PJIs. A CRP greater or equal to 82–100 mg/L within 6 weeks post‐operation is concerning for PJI, though not diagnostic [50, 51, 52, 75]. One should obtain a CBC with differential to look for systemic signs of infection and sepsis. As mentioned, ESR should not be used to evaluate for early PJI given poor utility in this period [80]. There is limited data on serum IL‐6 values for PJI specifically within 30 days of TJA; therefore, ordering this lab will be of limited utility. D‐dimer can remain elevated for 6 weeks or more following TJA, and it is recommended to avoid use in the early period due to nonspecificity [86, 87]. If there is clinical or laboratory suspicion of infection, then joint aspiration and synovial analysis should be performed [37]. Ultrasound‐guided joint aspiration is the most useful pre‐operative tool in evaluating for PJI [88]. Ideally, 3.5 mL of synovial fluid should be aspirated [59]. Synovial fluid should be sent for cell count with differential and culture [80]. Cultures should include aerobic and anaerobic cultures and should ideally be sent in blood culture bottles. Anaerobic bottles should be cultured for at least 14 days [85, 89]. Two positive cultures with the same organism are diagnostic for infection. The interpretation of a single positive culture depends upon the virulence of the organism identified. High virulence organisms such as *S. aureus* , aerobic gram‐negative bacilli, beta‐hemolytic streptococci, and *Enterococcus* spp. are common causes of early PJI and should have more diagnostic weight than organisms that may be contaminants, such as coagulase‐negative staphylococci or *C. acnes* [89, 90]. Single positive cultures should also be considered in the context of clinical and laboratory findings. If there is suspected S‐SSI or cellulitis near the incision site, then joint aspiration should be collected in a way where the needle avoids involved tissue [59, 91]. If there is no uninvolved skin surrounding the joint, then the decision for joint aspiration should be a risk‐vs‐benefit analysis. In this setting, aspiration should still be attempted in a patient who is septic or critically ill, but aspiration may be deferred to an orthopedic consult if the patient is stable.

In the emergency setting, cultures are unlikely to readily result. Instead, emergency providers should use synovial fluid analysis results to guide immediate decisions. For suspicion of early PJI, synovial cell count and differential should be evaluated at a higher threshold than those established for chronic PJI, given the inflammatory response in normal healing following TJA. Two studies mentioned above were selected to define synovial fluid thresholds for PJI in the early postoperative period. The systematic review and meta‐analysis found diagnostic odds ratios for PJI were 1468.99 and 111.74 for WBC thresholds ≥ 13,600/μL within 3 weeks and ≥ 9338/μL within 6 weeks of TJA, respectively. The same study also determined that the diagnostic odds ratio for PJI was 18.71 for both PMN values ≥ 90.5% within 3 weeks and ≥ 88% within 6 weeks of TJA [77]. An interval‐based study evaluated synovial WBC and differential at 0–15 days and 15–45 days following TJA. The optimal diagnostic threshold for PJI at 0–15 days were WBC count of 21,003 cells/μL (88.0% sensitivity, 100% specificity) and PMN% of 92% (56.0% sensitivity, 68.4% specificity). Subsequently, the optimal thresholds at 15–45 days were WBC count of 5111 cells/μL (90.5% sensitivity, 90.4% specificity) and PMN% of 80% (87.8% specificity and 88.5% sensitivity) [78]. Together these studies help establish that synovial WBC count and PMN percentage are highly time‐dependent in the early postoperative period, and applying chronic PJI thresholds during this timeframe would risk substantial diagnostic misclassification.

If synovial fluid cell count and differential is greater than the described thresholds for corresponding timeframes, then early PJI is likely. The orthopedic team should be alerted and treatment should be started. If values are clearly lower than these thresholds, then early PJI is unlikely. Emergency providers should reconsider the role of normal healing in the patients' presenting concerns. If clinical suspicion for infection remains high despite all values lower than the described thresholds, then clinical judgment should prompt orthopedic consultation. If values are inconclusive or borderline, orthopedic consultation is necessary and more testing, imaging, or intra‐operative evaluation may be performed. Rarely, more advanced imaging techniques (e.g., CT or MRI with metal reduction techniques) can be considered by the orthopedic team for complex presentations, though findings can be nonspecific and metal implants can interfere with imaging. Another imaging option includes indium‐111 labeled‐leucocyte scintigraphy and/or technetium‐99 m–labeled sulfur colloid bone marrow imaging [88]. These may be more appropriate for complex cases evaluating for chronic PJIs. Orthopedists may also perform synovial PCR or DNA sequencing in specific cases, however, these assays are not recommended for routine use by emergency providers in diagnosing early PJIs. Additionally, this technology may not be universally accessible.

Importantly, current criteria for diagnosing PJIs, such as the 2018 revised Musculoskeletal Infection Society (MSIS) criteria (also called International Consensus Meeting [ICM] criteria or Parvizi criteria) or the 2021 European Bone and Joint Infection Society (EBJIS) definition, lack validated data in specifically diagnosing early PJIs. Laboratory thresholds in the 2018 ICM/Parvizi criteria were developed and validated using data from chronic infections [92]. Similarly, the 2021 EBJIS definition was developed without differentiating the timeline of infection. This appropriately contextualizes that infection exists on a biologic continuum, but does not address the expectedly elevated inflammatory state following TJA [93]. Consequently, the laboratory thresholds used in these definitions can be misleading in the early postoperative period and may be much lower than expected for normal healing, as normal healing responses may exceed these thresholds established for chronic PJI. These criteria also require a battery of tests that may not be timely or readily accessible in the emergency setting [94]. Emergency providers should be aware of these criteria and their limitations in early PJI diagnosis. For completeness, the pre‐operative 2018 MSIS/ISM/Parvizi and 2021 EBJIS criteria are provided in supplemental resources. Below, we detail the differences from previous criteria and our algorithm in diagnosing PJIs (Figure 4).

**FIGURE 4:**
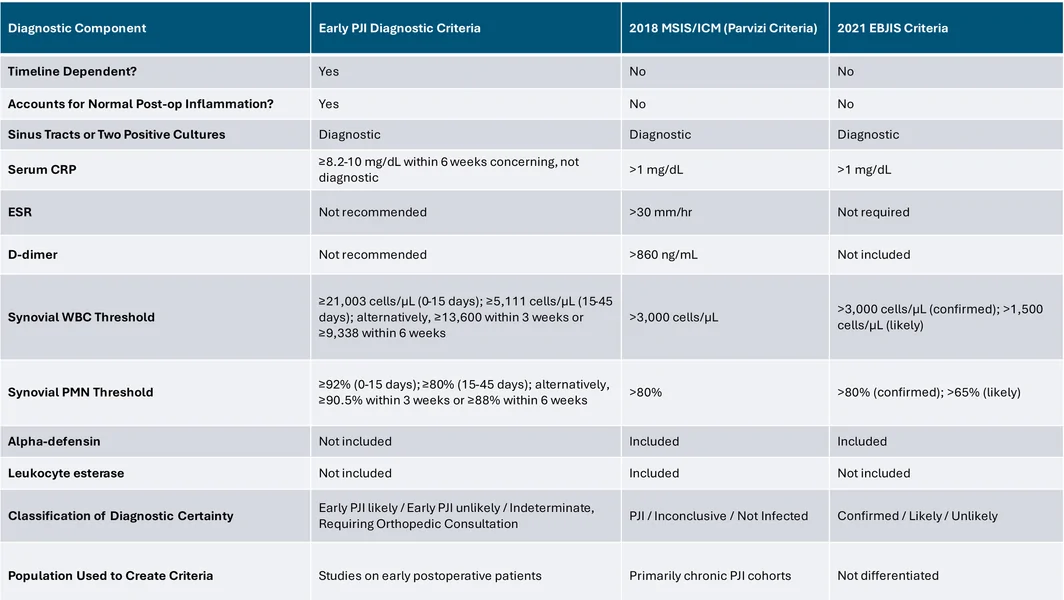
Comparison of our proposed diagnostic algorithm for question of early PJI compared to established criteria for undifferentiated PJI.

## 5 Principals of Management

In early postoperative TJA patients where infection has been ruled out and whose source of pain is normal inflammation, the guideline treatments of choice are COX‐2 selective NSAIDs and acetaminophen. COX‐2 inhibitors and acetaminophen limit opioid use and alleviate pain, while COX‐2 inhibition also improves function. It should be noted that oral acetaminophen is as effective as intravenous formulations in this population [95]. Several multimodal pain management protocols also add a proton pump inhibitor (PPI) for gastric protection [96, 97]. Nonpharmacologic methods for inflammation control can also be utilized to help patients control pain following TJA. Emergency providers can encourage postoperative techniques, such as early mobilization to a maximum of 750 steps/day in week 1, 1200 steps/day in week 2, 2000 steps/day in week 3, and 2750 steps/day in week 4 [34, 98]. Stairs should be limited in the first 2 weeks. Patients can also elevate and ice the target limb for 40 min per hour for the first 2 weeks, followed by 40 min at least 3 times/day for the next 2 weeks [34]. Emergency providers can also discuss pain expectations and realistic progression of gradual recovery with these patients, though it is acknowledged that this is the responsibility of the primary orthopedic team.

For patients with S‐SSI, incision and drainage (I&D) is the cornerstone of treatment, boasting success rates of 84% [38, 99]. Although providers commonly prescribe antibiotics for all superficial surgical site infections, there is limited evidence for this practice, as there is no difference in clinical outcome between I&D with antibiotics and I&D alone [38, 100, 101]. The ISDS recommends adding antibiotics if S‐SSI is accompanied by a temperature > 38.5°C, heart rate > 110 beats/min, or erythema extending > 5 cm beyond the incision site [38]. Additionally, the World Society of Emergency Surgery (WSES) and the Surgical Infection Society Europe (SIS‐E) recommend empiric antibiotics for S‐SSI with any SIRS criteria, signs of organ failure (hypotension, oliguria, decreased mental alertness), or in immunocompromised patients [61]. Empiric antibiotics or culture‐guided antibiotics can be prescribed for 24–48 h. First generation cephalosporins are empiric choices for suspicious S‐SSIs following clean arthroplasty procedures, as these infections are generally caused by *S. aureus* or streptococci. If MRSA risk factors exist (high proportion of MRSA infections in the institution where the operation was performed, patient with nasal colonization, patient with prior MRSA infection, patient previously on antibiotics, pre‐operative MRSA screen positive), then an antibiotic with MRSA coverage should be given (vancomycin, linezolid, daptomycin, telavancin, or ceftaroline) along with an antibiotic covering gram‐negatives and anaerobes (piperacillin‐tazobactam, a carbapenem, or ceftriaxone with metronidazole) [38]. A stable patient with S‐SSI may be discharged with 24–48 h of cephalexin alone (no MRSA risk factors) or cephalexin with TMP‐SMX or doxycycline, or clindamycin alone (MRSA risk factors present). An unstable patient with S‐SSI should receive orthopedic consultation for treatment disposition. If cellulitis is suspected, antibiotics should be given for 5 days [38]. Empiric choices for cellulitis near the site of incision should be vancomycin if the patient is immunocompetent and without systemic signs of infection; patients can then be transitioned to outpatient oral cephalexin if without MRSA risk factors. If MRSA risk factors are present, patients should be prescribed outpatient cephalexin with TMP‐SMX or doxycycline, or clindamycin alone. If the patient with suspected cellulitis shows systemic signs of infection or is immunocompromised, then the patient should be started on vancomycin and piperacillin‐tazobactam and admitted for treatment [38]. Importantly, joint aspiration for suspicion of PJI should be performed before antibiotics are given unless the patient has signs of sepsis, as antibiotics can increase rates of false negative cultures [80, 85, 102].

For early PJI, institutional protocols may guide treatment. Debridement with antibiotics and implant retention (DAIR) is generally the treatment of choice unless joint arthroplasty revision surgery is required [59]. Following aspiration, the orthopedic team should be consulted for disposition recommendations and aid in management. Depending on institution workflow, emergency providers may be responsible for starting empiric antibiotics, though antibiotics may still be deferred after aspiration if the patient is clinically stable. In that instance, there should be prompt discussion with the orthopedic team on whether empiric antibiotics should be started before or after intraoperative surgical exploration with tissue sampling [85]. Empiric antibiotic choice is generally vancomycin with a broad‐spectrum beta‐lactam, such as piperacillin‐tazobactam or a third‐generation cephalosporin, though there is an increased risk of kidney injury with vancomycin and piperacillin‐tazobactam compared to vancomycin with the cephalosporin [103]. It is also important to note that in sepsis patients, vancomycin with piperacillin‐tazobactam has a statistically higher risk of 90‐day mortality compared to vancomycin with the 4th generation cephalosporin, cefepime [104].

Notable risk factors should be taken into consideration for management. Diabetes has been found to nearly double the risk of PJI [105]. Furthermore, obesity may impair antibiotic penetration [106]. Immunocompromised patients may have attenuated clinical signs of infection [107]. Prior PJI is a strong risk factor for PJI, and prior MRSA history/colonization is a risk factor for both S‐SSI and PJI [108, 109]. While the overall algorithm steps do not change significantly for these patients, the above‐mentioned risk factors should lower thresholds for testing, empiric antibiotic treatment, and broader antibiotic choices.

Upon discharge, patients should ideally visit their orthopedist within 2 days. If cultures have been performed, preliminary results may be available. Alternatively, orthopedists may use newer technology, such as synovial PCR or DNA sequencing to detect infection. Unlike traditional culturing, these tools are still able to identify infection despite antibiotic usage. However, limitations do exist such as cost, access, and turn‐around time. Although this emerging technology is promising, there is currently a limited role for them in the emergency setting.

## 6 Limitations and Future Directions

This proposed early PJI diagnostic algorithm has several limitations. Our algorithm represents an evidence‐informed framework synthesized from the available literature that has not been prospectively validated. The criteria in this algorithm have been mostly derived from retrospective, single‐center studies with a small number of early infections; these studies have also had heterogeneity in patient populations, aspiration timing, technique, and lab methods. The current research on early TJA infections is limited compared to data on subacute and chronic infections, which further reduces available knowledge on this topic. Furthermore, some resources (i.e., orthopedic consultation, ultrasound‐guided aspiration) may not be available in all emergency settings, and institutional protocols may differ from our proposals. Our algorithm should complement and not replace emergency provider judgment and orthopedic consultation. Future research should focus on prospective validation across multiple centers and should attempt external validation across academic, community, and rural settings. There should be comparison between our novel algorithm and previous PJI diagnostic criteria, determining sensitivity, specificity, and predictive values.

## 7 Conclusion

Emergency department evaluation of patients in the early period following total joint arthroplasty is increasingly common and may be confounded by normal postoperative inflammation that can mimic infection. These overlapping features may prompt defensive medical practices despite the low incidence of early periprosthetic joint infection, leading to increased healthcare costs. This phenomenon is especially important under the Transforming Episode Accountability Model (TEAM), where all care related to TJA within 30 days of surgery is bundled into a single payment that directly links ED resource utilization to institutional financial performance. By emphasizing a deep understanding of clinical presentation during normal healing and by using a time‐dependent interpretation of laboratory values, emergency providers can reduce low‐value care while still maintaining patient safety. Adoption of a standardized diagnostic algorithm will allow for efficient, safe, and high‐value care that is medicolegally defensible in the current emergency medicine landscape.

## Author Contributions

**Thomas W. Brown:** conceptualization, literature review, data collection, manuscript drafting, figure creation. **Andrew T. Carusi:** literature review, manuscript editing, figure creation. **Michael Langworthy:** supervision, clinical expertise, manuscript review and revision.

## Funding

The authors have nothing to report.

## Ethics Statement

This study involved literature review and analysis of publicly available hospital pricing data. It did not involve human subjects. Institutional Review Board approval was not required.

## Conflicts of Interest

The authors declare no conflicts of interest.

## Supporting information


**Data S1:** Supporting Information.

**Data S2:** Supporting Information.

## Data Availability

The data that support the findings of this study are openly available in Southcoast Health Charge Masters at https://www.southcoast.org/southcoast‐health‐care‐charges/.

## References

[1] M. Zabaglo , S. W. Leslie , and T. Sharman , “Postoperative Wound Infections,” StatPearls (2025): NBK560533. 32809368

[2] A. J. Tande and R. Patel , “Prosthetic Joint Infection,” Clinical Microbiology Reviews 27, no. 2 (2014): 302–345, 10.1128/CMR.00111-13. PMC3993098 24696437

[3] H. K. Eriksson and S. Lazarinis , “Patient‐Related Factors Associated With Superficial Surgical Site Infection and Progression to a Periprosthetic Joint Infection After Elective Primary Total Joint Arthroplasty: A Single‐Centre, Retrospective Study in Sweden,” BMJ Open 12, no. 9 (2022): e060754, 10.1136/bmjopen-2022-060754. PMC9486361 36123083

[4] B. H. Kapadia , R. A. Berg , J. A. Daley , J. Fritz , A. Bhave , and M. A. Mont , “Periprosthetic Joint Infection,” Lancet 387, no. 10016 (2016): 386–394, 10.1016/S0140-6736(14)61798-0. 26135702

[5] B. Fink , M. Hoyka , E. Weissbarth , P. Schuster , and I. Berger , “A New Graphic Type Differentiation of Cell Account Determination for Distinguishing Acute Periprosthetic Joint Infection From Hemarthrosis,” Antibiotics 11, no. 10 (2022): 1284, 10.3390/antibiotics11101284. PMC9598683 36289943

[6] D. S. Phillips , K. K. Workman , and M. Kelly , “Nonoperative Treatment of a Periprosthetic Gout Flare in the Setting of a Positive Alpha‐Defensin Result,” Arthroplast Today 9 (2021): 65–67, 10.1016/j.artd.2021.04.007. PMC8141601 34041331

[7] M. A. Incesoy , C. B. Demirkiran , H. B. Kaya , et al., “Natural Course of Postoperative C‐Reactive Protein and Erythrocyte Sedimentation Rate in Unilateral and Simultaneous Bilateral Total Knee Arthroplasty,” BMC Musculoskeletal Disorders 26, no. 1 (2025): 260, 10.1186/s12891-025-08523-0. PMC11907866 40087715

[8] Y. S. Choi , J. B. Oh , M. J. Chang , T. W. Kim , K. S. Kang , and S. Kang , “Delayed Normalization of C‐Reactive Protein and Erythrocyte Sedimentation Rate Was Not Associated With Inferior Clinical Outcomes After Total Knee Arthroplasty,” Journal of Orthopaedic Science 28, no. 3 (2023): 589–596, 10.1016/j.jos.2022.02.007. 35331605

[9] R. R. Choudhry , R. P. Rice , P. D. Triffitt , W. M. Harper , and P. J. Gregg , “Plasma Viscosity and C‐Reactive Protein After Total Hip and Knee Arthroplasty,” Journal of Bone and Joint Surgery. British Volume 74, no. 4 (1992): 523–524, 10.1302/0301-620X.74B4.1624508. 1624508

[10] I. B. Molloy , B. I. Martin , W. E. Moschetti , and D. S. Jevsevar , “Effects of the Length of Stay on the Cost of Total Knee and Total Hip Arthroplasty From 2002 to 2013,” Journal of Bone and Joint Surgery (American Volume) 99, no. 5 (2017): 402–407, 10.2106/JBJS.16.00019. PMC5324037 28244911

[11] C. J. Carr , S. C. Mears , C. L. Barnes , and J. B. Stambough , “Length of Stay After Joint Arthroplasty Is Less Than Predicted Using Two Risk Calculators,” Journal of Arthroplasty 36, no. 9 (2021): 3073–3077, 10.1016/j.arth.2021.04.010. PMC8380646 33933330

[12] R. Papalia , G. Torre , A. M. Alifano , et al., “Length of Hospital Stay After Total Knee Arthroplasty: A Correlation Study on 1200 Patients,” Journal of Clinical Medicine 11, no. 8 (2022): 2114, 10.3390/jcm11082114. PMC9029058 35456209

[13] M. Loppini , F. M. Gambaro , R. Nelissen , and G. Grappiolo , “Large Variation in Timing of Follow‐Up Visits After Hip Replacement: A Review of the Literature,” EFORT Open Reviews 7, no. 3 (2022): 200–205, 10.1530/EOR-21-0016. PMC8965200 35298395

[14] A. H. Stone , L. Dunn , J. H. MacDonald , and P. J. King , “Reducing Length of Stay Does Not Increase Emergency Room Visits or Readmissions in Patients Undergoing Primary Hip and Knee Arthroplasties,” Journal of Arthroplasty 33, no. 8 (2018): 2381–2386, 10.1016/j.arth.2018.03.043. 29656979

[15] U. S. Sibia , A. E. Mandelblatt , M. A. Callanan , J. H. MacDonald , and P. J. King , “Incidence, Risk Factors, and Costs for Hospital Returns After Total Joint Arthroplasties,” Journal of Arthroplasty 32, no. 2 (2017): 381–385, 10.1016/j.arth.2016.08.003. 27597429

[16] M. Langworthy , “Use of a Novel, Extended‐Release, Dual‐Acting, Local Anesthetic in Total Knee Arthroplasty and Total Hip Arthroplasty: Application Technique and Experiences With ZYNRELEF,” Journal of Orthopaedic Experience & Innovation 5, no. 1 (2024): 90728, 10.60118/001c.90728.

[17] A. D. Kaye , A. N. Edinoff , J. Y. Yan , et al., “Novel Local Anesthetics in Clinical Practice: Pharmacologic Considerations and Potential Roles for the Future,” Anesthesia and Pain Medicine 12, no. 1 (2022): e123112, 10.5812/aapm.123112. PMC8995776 35433373

[18] T. Ottoboni , B. Quart , J. Pawasauskas , J. F. Dasta , R. A. Pollak , and E. R. Viscusi , “Mechanism of Action of HTX‐011: A Novel, Extended‐Release, Dual‐Acting Local Anesthetic Formulation for Postoperative Pain,” Regional Anesthesia and Pain Medicine 45 (2019): 117–123, 10.1136/rapm-2019-100714. 31843865

[19] B. J. Loyd , A. J. Kittelson , J. Forster , S. Stackhouse , and J. Stevens‐Lapsley , “Development of a Reference Chart to Monitor Postoperative Swelling Following Total Knee Arthroplasty,” Disability and Rehabilitation 42, no. 12 (2020): 1767–1774, 10.1080/09638288.2018.1534005. 30668214

[20] Centers for Medicare & Medicaid Services, Transforming Episode Accountability Model (TEAM) , US Centers for Medicare & Medicaid Services (2025). 10.1097/DCR.0000000000004286 42170854

[21] K. M. Sauro , C. Smith , S. Ibadin , et al., “Enhanced Recovery After Surgery Guidelines and Hospital Length of Stay, Readmission, Complications, and Mortality: A Meta‐Analysis of Randomized Clinical Trials,” JAMA Network Open 7, no. 6 (2024): e2417310, 10.1001/jamanetworkopen.2024.17310. PMC11195621 38888922

[22] W. B. Bauerle , S. Hamlin , S. Dubois , et al., “Impact of Liposomal Bupivacaine on Enhanced Recovery After Surgery Protocol for Lung Resection,” Annals of Thoracic Surgery 119, no. 1 (2025): 219–226, 10.1016/j.athoracsur.2024.05.015. 38815849

[23] K. Candiotti , “Liposomal Bupivacaine: An Innovative Nonopioid Local Analgesic for the Management of Postsurgical Pain,” Pharmacotherapy 32, no. 9 Suppl (2012): 19S–26S, 10.1002/j.1875-9114.2012.01183.x. 22956491

[24] M. P. Kelly , H. A. Prentice , W. Wang , B. H. Fasig , D. S. Sheth , and E. W. Paxton , “Reasons for Ninety‐Day Emergency Visits and Readmissions After Elective Total Joint Arthroplasty: Results From a US Integrated Healthcare System,” Journal of Arthroplasty 33, no. 7 (2018): 2075–2081, 10.1016/j.arth.2018.02.010. 29523446

[25] N. Maldonado‐Rodriguez , S. Ekhtiari , M. M. Khan , et al., “Emergency Department Presentation After Total Hip and Knee Arthroplasty: A Systematic Review,” Journal of Arthroplasty 35, no. 10 (2020): 3038–3045.e1, 10.1016/j.arth.2020.05.022. 32540306

[26] M. Schindler , S. Schmitz , J. Reinhard , P. Jansen , J. Grifka , and A. Benditz , “Pain Course After Total Knee Arthroplasty Within a Standardized Pain Management Concept: A Prospective Observational Study,” Journal of Clinical Medicine 11, no. 23 (2022): 7204, 10.3390/jcm11237204. PMC9741301 36498779

[27] F. Riyahi , S. Riahy , and M. Yousefpour , “Reviewing the Physiology of Cutaneous Wound Healing and Evaluating the Effect of Exercise on It: A Narrative Review Article,” Annals of Military and Health Sciences Research 19, no. 3 (2021): e115926, 10.5812/amh.115926.

[28] O. Dada , A. Gonzalez Zacarias , C. Ongaigui , et al., “Does Rebound Pain After Peripheral Nerve Block for Orthopedic Surgery Impact Postoperative Analgesia and Opioid Consumption? A Narrative Review,” International Journal of Environmental Research and Public Health 16, no. 18 (2019): 3257, 10.3390/ijerph16183257. PMC6765957 31491863

[29] B. M. Admassie , S. A. Debas , and B. A. Admass , “Prevention and Management of Rebound Pain After Resolution of Regional Block: A Systematic Review,” Annals of Medicine and Surgery 86, no. 8 (2024): 4732–4737, 10.1097/MS9.0000000000002299. PMC11305791 39118752

[30] E. M. Pogatzki‐Zahn , D. Segelcke , and S. A. Schug , “Postoperative Pain‐From Mechanisms to Treatment,” Pain Reports 2, no. 2 (2017): e588, 10.1097/PR9.0000000000000588. PMC5770176 29392204

[31] X. X. Chen , T. Wang , J. Li , and H. Kang , “Relationship Between Inflammatory Response and Estimated Complication Rate After Total Hip Arthroplasty,” Chinese Medical Journal (English Edition) 129, no. 21 (2016): 2546–2551, 10.4103/0366-6999.192772. PMC5125332 27779160

[32] D. Ovadia , E. Luger , J. Bickels , A. Menachem , and S. Dekel , “Efficacy of Closed Wound Drainage After Total Joint Arthroplasty. A Prospective Randomized Study,” Journal of Arthroplasty 12, no. 3 (1997): 317–321, 10.1016/s0883-5403(97)90029-2. 9113547

[33] G. R. Scuderi , “Avoiding Postoperative Wound Complications in Total Joint Arthroplasty,” Journal of Arthroplasty 33, no. 10 (2018): 3109–3112, 10.1016/j.arth.2018.01.025. 29475573

[34] A. Wickline , W. Cole , M. Melvin , S. Ehmann , F. Aviles , and J. Bradt , “Mitigating the Post‐Operative Swelling Tsunami in Total Knee Arthroplasty: A Call to Action,” Journal of Orthopaedic Experience & Innovation 4, no. 2 (2023): 77444, 10.60118/001c.77444.

[35] Y. H. Pua , “The Time Course of Knee Swelling Post Total Knee Arthroplasty and Its Associations With Quadriceps Strength and Gait Speed,” Journal of Arthroplasty 30, no. 7 (2015): 1215–1219, 10.1016/j.arth.2015.02.010. 25737387

[36] L. Gavish , L. Kandel , G. Rivkin , S. D. Gertz , and O. Hoffer , “Natural History of Changes in Knee Skin Temperature Following Total Knee Arthroplasty: A Systematic Review and Meta‐Analysis,” Scientific Reports 13, no. 1 (2023): 6810, 10.1038/s41598-023-33556-7. PMC10133306 37100814

[37] a. Expert Panel on Musculoskeletal , E. A. Walker , M. G. Fox , et al., “ACR Appropriateness Criteria(R) Imaging After Total Knee Arthroplasty: 2023 Update,” Journal of the American College of Radiology 20, no. 11S (2023): S433–S454, 10.1016/j.jacr.2023.08.014. 38040463

[38] D. L. Stevens , A. L. Bisno , H. F. Chambers , et al., “Practice Guidelines for the Diagnosis and Management of Skin and Soft Tissue Infections: 2014 Update by the Infectious Diseases Society of America,” Clinical Infectious Diseases 59, no. 2 (2014): e10–e52, https://academic.oup.com/cid/article/59/2/e10/2895845. 10.1093/cid/ciu444 24973422

[39] S. Akil , J. M. Newman , N. V. Shah , N. Ahmed , A. J. Deshmukh , and A. V. Maheshwari , “Metal Hypersensitivity in Total Hip and Knee Arthroplasty: Current Concepts,” Journal of Clinical Orthopaedics and Trauma 9, no. 1 (2018): 3–6, 10.1016/j.jcot.2017.10.003. PMC5884053 29628676

[40] E. Jolkovsky , E. Zeegen , and I. Ahronowitz , “Eczematous Neurodermatitis Following Total Knee Arthroplasty: Expanding Awareness of a Rare and Underdiagnosed Postoperative Dermatologic Complication,” Cureus 17, no. 6 (2025): e86989, 10.7759/cureus.86989. PMC12307027 40734875

[41] M. Nazeer , R. Ravindran , B. C. Katragadda , E. N. Muhammed , D. T. J. Rema , and M. N. Muhammed , “SKINTED: A Rare Complication After Total Knee Arthroplasty,” Arthroplast Today 6, no. 4 (2020): 1028–1032, 10.1016/j.artd.2020.10.004. PMC7772457 33385046

[42] L. Wuyts , F. Van Dyck , N. Kamerling , et al., “Reticular Telangiectatic Erythema From Implanted Devices Attributed to Low‐Grade Infection With *Staphylococcus epidermidis* ,” Medical Devices & Sensors 2, no. 1 (2019): e10023, 10.1002/mds3.10023.

[43] H. K. Do and N. Mousdicas , “Reticular Telangiectatic Erythema After Total Knee Replacement Surgery: A Report of Two Cases,” JBJS Case Connector 3, no. 2 (2013): e49, 10.2106/JBJS.CC.L.00193. 29252401

[44] M. J. Langworthy , K. Hankins , and T. Storey , “The Basic and Clinical Science of Wounds in the Austere Environment,” Journal of Orthopaedic Experience & Innovation 4, no. 2 (2023): 75162, 10.60118/001c.75162.

[45] S. Larsson , U. Thelander , and S. Friberg , “C‐Reactive Protein (CRP) Levels After Elective Orthopedic Surgery,” Clinical Orthopaedics and Related Research 275 (1992): 237–242. 1735220

[46] J. White , M. Kelly , and R. Dunsmuir , “C‐Reactive Protein Level After Total Hip and Total Knee Replacement,” Journal of Bone and Joint Surgery. British Volume (London) 80, no. 5 (1998): 909–911, 10.1302/0301-620x.80b5.8708. 9768908

[47] R. O. Niskanen , O. Korkala , and H. Pammo , “Serum C‐Reactive Protein Levels After Total Hip and Knee Arthroplasty,” Journal of Bone and Joint Surgery. British Volume 78, no. 3 (1996): 431–433. 8636181

[48] Z. Y. Huang , Q. Huang , L. Y. Wang , et al., “Normal Trajectory of Interleukin‐6 and C‐Reactive Protein in the Perioperative Period of Total Knee Arthroplasty Under an Enhanced Recovery After Surgery Scenario,” BMC Musculoskeletal Disorders 21, no. 1 (2020): 264, 10.1186/s12891-020-03283-5. PMC7175526 32316949

[49] K. Nazem , M. Motififard , and M. Yousefian , “Variations in ESR and CRP in Total Knee Arthroplasty and Total Hip Arthroplasty in Iranian Patients From 2009 to 2011,” Advanced Biomedical Research 5 (2016): 148, 10.4103/2277-9175.187403. PMC5025920 27656617

[50] P. H. Yi , M. B. Cross , M. Moric , S. M. Sporer , R. A. Berger , and C. J. Della Valle , “The 2013 Frank Stinchfield Award: Diagnosis of Infection in the Early Postoperative Period After Total Hip Arthroplasty,” Clinical Orthopaedics and Related Research 472, no. 2 (2014): 424–429, 10.1007/s11999-013-3089-1. PMC3890203 23884798

[51] E. M. Dugdale , M. E. Uvodich , D. R. Osmon , M. W. Pagnano , D. J. Berry , and M. P. Abdel , “Laboratory Value Effectiveness in Predicting Early Postoperative Periprosthetic Joint Infection After Total Hip Arthroplasty,” Journal of Arthroplasty 37, no. 3 (2022): 574–580, 10.1016/j.arth.2021.11.007. 34774687

[52] H. Bedair , N. Ting , C. Jacovides , et al., “The Mark Coventry Award: Diagnosis of Early Postoperative TKA Infection Using Synovial Fluid Analysis,” Clinical Orthopaedics and Related Research 469, no. 1 (2011): 34–40, 10.1007/s11999-010-1433-2. PMC3008895 20585914

[53] G. K. Deirmengian , B. Zmistowski , C. Jacovides , J. O'Neil , and J. Parvizi , “Leukocytosis Is Common After Total Hip and Knee Arthroplasty,” Clinical Orthopaedics and Related Research 469, no. 11 (2011): 3031–3036, 10.1007/s11999-011-1887-x. PMC3183208 21461866

[54] C. P. Christensen , H. Bedair , C. J. Della Valle , J. Parvizi , B. Schurko , and C. A. Jacobs , “The Natural Progression of Synovial Fluid White Blood‐Cell Counts and the Percentage of Polymorphonuclear Cells After Primary Total Knee Arthroplasty: A Multicenter Study,” Journal of Bone and Joint Surgery. American Volume 95, no. 23 (2013): 2081–2087, 10.2106/JBJS.L.01646. 24306694

[55] S. R. Brannan and D. A. Jerrard , “Synovial Fluid Analysis,” Journal of Emergency Medicine 30, no. 3 (2006): 331–339, 10.1016/j.jemermed.2005.05.029. 16677989

[56] M. Maritati , A. Trentini , D. Chemello , et al., “Predictive Factors of Surgical Site Infection in Prosthetic Joint Surgery: A Prospective Study on 760 Arthroplasties,” Mediators of Inflammation 2022 (2022): 2150804, 10.1155/2022/2150804. PMC9532124 36204658

[57] U. P. Dior , S. Kathurusinghe , C. Cheng , et al., “Effect of Surgical Skin Antisepsis on Surgical Site Infections in Patients Undergoing Gynecological Laparoscopic Surgery: A Double‐Blind Randomized Clinical Trial,” JAMA Surgery 155, no. 9 (2020): 807–815, 10.1001/jamasurg.2020.1953. PMC7344832 32639545

[58] A. S. Beydoun , K. Koss , T. Nielsen , et al., “Perioperative Topical Antisepsis and Surgical Site Infection in Patients Undergoing Upper Aerodigestive Tract Reconstruction,” JAMA Otolaryngology–Head & Neck Surgery 148, no. 6 (2022): 547–554, 10.1001/jamaoto.2022.0684. PMC9047735 35476816

[59] R. Patel , “Periprosthetic Joint Infection,” New England Journal of Medicine 388, no. 3 (2023): 251–262, 10.1056/NEJMra2203477. 36652356

[60] Centers for Diseases Control and Prevention, National Healthcare Safety Network (NHSN) , Outpatient Procedure Component Manual (Centers for Disease Control and Prevention) (2026), 1–61.

[61] M. Sartelli , X. Guirao , T. C. Hardcastle , et al., “2018 WSES/SIS‐E Consensus Conference: Recommendations for the Management of Skin and Soft‐Tissue Infections,” World Journal of Emergency Surgery: WJES 13 (2018): 58, 10.1186/s13017-018-0219-9. PMC6295010 30564282

[62] N. Sodhi , H. K. Anis , A. J. Acuna , et al., “Early Infection Trends for Primary Total Knee Arthroplasty: A 5‐Year Review,” Journal of Knee Surgery 36, no. 3 (2023): 269–273, 10.1055/s-0041-1731736. 34261159

[63] X. Jin , B. Gallego Luxan , M. Hanly , et al., “Estimating Incidence Rates of Periprosthetic Joint Infection After Hip and Knee Arthroplasty for Osteoarthritis Using Linked Registry and Administrative Health Data,” Bone & Joint Journal 104‐B, no. 9 (2022): 1060–1066, 10.1302/0301-620X.104B9.BJJ-2022-0116.R1. PMC9948458 36047015

[64] S. Asadollahi , T. W. Hamilton , S. A. Sabah , et al., “The Outcomes of Acute Periprosthetic Joint Infection Following Unicompartmental Knee Replacement Managed With Early Debridement, Antibiotics, and Implant Retention,” Knee 47 (2024): 13–20, 10.1016/j.knee.2023.12.001. 38171207

[65] E. J. Weinstein , A. J. Stephens‐Shields , C. W. Newcomb , et al., “Incidence, Microbiological Studies, and Factors Associated With Prosthetic Joint Infection After Total Knee Arthroplasty,” JAMA Network Open 6, no. 10 (2023): e2340457, 10.1001/jamanetworkopen.2023.40457. PMC10618849 37906194

[66] W. Zimmerli , A. Trampuz , and P. E. Ochsner , “Prosthetic‐Joint Infections,” New England Journal of Medicine 351, no. 16 (2004): 1645–1654, 10.1056/NEJMra040181. 15483283

[67] N. Shohat , K. Goswami , T. L. Tan , et al., “Fever and Erythema Are Specific Findings in Detecting Infection Following Total Knee Arthroplasty,” Journal of Bone and Joint Infection 4, no. 2 (2019): 92–98, 10.7150/jbji.30088. PMC6470654 31011514

[68] T. N. Peel , A. C. Cheng , K. L. Buising , and P. F. Choong , “Microbiological Aetiology, Epidemiology, and Clinical Profile of Prosthetic Joint Infections: Are Current Antibiotic Prophylaxis Guidelines Effective?,” Antimicrobial Agents and Chemotherapy 56, no. 5 (2012): 2386–2391, 10.1128/AAC.06246-11. PMC3346661 22314530

[69] P. Brumat , B. Mavcic , I. Jurcic , and R. Trebse , “Clinical and Laboratory Predictors for Prosthetic Joint Infection Within the First Postoperative Days Following Primary Total Hip and Knee Arthroplasty,” International Orthopaedics 47, no. 9 (2023): 2173–2179, 10.1007/s00264-023-05891-x. PMC10439017 37421426

[70] Y. Ishii , H. Noguchi , M. Takeda , J. Sato , S. Takayama , and S. Toyabe , “Characteristics and Significance of Fever During 4 Weeks After Primary Total Knee Arthroplasty,” Archives of Orthopaedic and Trauma Surgery 134, no. 5 (2014): 707–712, 10.1007/s00402-014-1949-0. PMC3990857 24522863

[71] J. L. Seidelman , C. R. Mantyh , and D. J. Anderson , “Surgical Site Infection Prevention: A Review,” Journal of the American Medical Association 329, no. 3 (2023): 244–252, 10.1001/jama.2022.24075. 36648463

[72] M. Budin , T. D. Luo , C. Lausmann , T. Gehrke , and M. Citak , “What Are the Risk Factors and Microorganism Profiles of Periprosthetic Hip Joint Infections With a Concomitant Sinus Tract?,” Archives of Orthopaedic and Trauma Surgery 145, no. 1 (2024): 84, 10.1007/s00402-024-05713-8. 39708188

[73] T. D. Luo , M. Budin , T. Karlidag , C. Lausmann , T. Gehrke , and M. Citak , “Risk Factors and Microbiological Profile of Knee Periprosthetic Joint Infections With Sinus Tract,” Journal of Arthroplasty 40, no. 1 (2025): 214–217, 10.1016/j.arth.2024.06.062. 38969295

[74] C. Xu , Q. Wang , F. C. Kuo , K. Goswami , T. L. Tan , and J. Parvizi , “The Presence of Sinus Tract Adversely Affects the Outcome of Treatment of Periprosthetic Joint Infections,” Journal of Arthroplasty 34, no. 6 (2019): 1227–1232.e2, 10.1016/j.arth.2019.02.040. 30905639

[75] M. E. Uvodich , E. M. Dugdale , D. R. Osmon , M. W. Pagnano , D. J. Berry , and M. P. Abdel , “The Effectiveness of Laboratory Tests to Predict Early Postoperative Periprosthetic Infection After Total Knee Arthroplasty,” Bone & Joint Journal 103‐B, no. 6 Supple A (2021): 177–184, 10.1302/0301-620X.103B6.BJJ-2020-2397.R1. 34053291

[76] S. G. Kim , J. G. Kim , K. M. Jang , S. B. Han , H. C. Lim , and J. H. Bae , “Diagnostic Value of Synovial White Blood Cell Count and Serum C‐Reactive Protein for Acute Periprosthetic Joint Infection After Knee Arthroplasty,” Journal of Arthroplasty 32, no. 12 (2017): 3724–3728, 10.1016/j.arth.2017.07.013. 28800858

[77] M. Sabater‐Martos , M. Ferrer , L. Morata , A. Soriano , and J. C. Martinez‐Pastor , “Diagnostic Cutoff Values of Synovial Fluid Biomarkers for Acute Postoperative Prosthetic Joint Infection: A Systematic Review and Meta‐Analysis,” Journal of Bone and Joint Infection 9, no. 1 (2024): 17–26, 10.5194/jbji-9-17-2024. PMC11002915 38601003

[78] S. Tarabichi , J. T. Verhey , J. D. Lizcano , et al., “Diagnostic Thresholds of Synovial Markers for Acute Periprosthetic Joint Infection: One Size Does Not Fit All,” Journal of Arthroplasty 40, no. 7S1 (2025): S296–S302, 10.1016/j.arth.2025.02.064. 40023460

[79] K. Sukhonthamarn , T. L. Tan , C. Xu , et al., “Determining Diagnostic Thresholds for Acute Postoperative Periprosthetic Joint Infection,” Journal of Bone and Joint Surgery. American Volume 102, no. 23 (2020): 2043–2048, 10.2106/JBJS.20.00257. 32941311

[80] T. A. Luthringer , Y. A. Fillingham , K. Okroj , E. J. Ward , and C. Della Valle , “Periprosthetic Joint Infection After Hip and Knee Arthroplasty: A Review for Emergency Care Providers,” Annals of Emergency Medicine 68, no. 3 (2016): 324–334, 10.1016/j.annemergmed.2016.03.004. 27083857

[81] S. Baertl , D. Lovasz , M. G. Kees , et al., “Periprosthetic Joint Infection and Concomitant Sepsis: Unveiling Clinical Manifestations, Risk Factors, and Patient Outcomes,” Journal of Arthroplasty 40, no. 7 (2025): 1827–1835, 10.1016/j.arth.2024.11.062. 39709100

[82] L. Ludwick , M. Siqueira , N. Shohat , M. B. Sherman , S. Streicher , and J. Parvizi , “For Patients With Acute PJI Treated With Debridement, Antibiotics, and Implant Retention, What Factors Are Associated With Systemic Sepsis and Recurrent or Persistent Infection in Septic Patients?,” Clinical Orthopaedics and Related Research 480, no. 8 (2022): 1491–1500, 10.1097/CORR.0000000000002192. PMC9278913 35420556

[83] S. Hofmann , G. Seitlinger , O. Djahani , and M. Pietsch , “The Painful Knee After TKA: A Diagnostic Algorithm for Failure Analysis,” Knee Surgery, Sports Traumatology, Arthroscopy 19, no. 9 (2011): 1442–1452, 10.1007/s00167-011-1634-6. 21822665

[84] Expert Panel on Musculoskeletal I , B. N. Weissman , C. J. Palestro , et al., “ACR Appropriateness Criteria(R) Imaging After Total Hip Arthroplasty,” Journal of the American College of Radiology 20, no. 11S (2023): S413–S432, 10.1016/j.jacr.2023.08.015. 38040462

[85] C. C. Tubb , G. G. Polkowksi , and B. Krause , “Diagnosis and Prevention of Periprosthetic Joint Infections,” Journal of the American Academy of Orthopaedic Surgeons 28, no. 8 (2020): e340–e348, 10.5435/JAAOS-D-19-00405. 31972719

[86] I. Azboy , B. Catal , K. Basarir , M. Mutlu , O. F. Bilgen , and J. Parvizi , “The Natural Course of Serum D‐Dimer, C‐Reactive Protein, and Erythrocyte Sedimentation Rate Levels After Uneventful Primary Total Joint Arthroplasty,” Journal of Arthroplasty 36, no. 9 (2021): 3118–3122, 10.1016/j.arth.2021.04.031. 34088567

[87] T. J. An , S. M. Engstrom , W. K. Oelsner , M. A. Benvenuti , G. G. Polkowski , and J. G. Schoenecker , “Elevated d‐Dimer Is Not Predictive of Symptomatic Deep Venous Thrombosis After Total Joint Arthroplasty,” Journal of Arthroplasty 31, no. 10 (2016): 2269–2272, 10.1016/j.arth.2016.02.059. 27062350

[88] A. Del Arco and M. L. Bertrand , “The Diagnosis of Periprosthetic Infection,” Open Orthopaedics Journal 7 (2013): 178–183, 10.2174/1874325001307010178. PMC3722546 23898349

[89] J. M. Miller , M. J. Binnicker , S. Campbell , et al., “Guide to Utilization of the Microbiology Laboratory for Diagnosis of Infectious Diseases: 2024 Update by the Infectious Diseases Society of America (IDSA) and the American Society for Microbiology (ASM),” Clinical Infectious Diseases (2024): ciae104, 10.1093/cid/ciae104. 38442248

[90] F. Ayoade , D. Li , A. Mabrouk , and J. R. Todd , “Periprosthetic Joint Infection,” StatPearls (2025): NBK448131. 28846340

[91] M. E. Margaretten , J. Kohlwes , D. Moore , and S. Bent , “Does This Adult Patient Have Septic Arthritis?,” Journal of the American Medical Association 297, no. 13 (2007): 1478–1488, 10.1001/jama.297.13.1478. 17405973

[92] J. Parvizi , T. L. Tan , K. Goswami , et al., “The 2018 Definition of Periprosthetic Hip and Knee Infection: An Evidence‐Based and Validated Criteria,” Journal of Arthroplasty 33, no. 5 (2018): 1309–1314.e2, 10.1016/j.arth.2018.02.078. 29551303

[93] M. McNally , R. Sousa , M. Wouthuyzen‐Bakker , et al., “The EBJIS Definition of Periprosthetic Joint Infection,” Bone & Joint Journal 103‐B, no. 1 (2021): 18–25, 10.1302/0301-620X.103B1.BJJ-2020-1381.R1. PMC7954183 33380199

[94] S. J. Kim and Y. J. Cho , “Current Guideline for Diagnosis of Periprosthetic Joint Infection: A Review Article,” Hip & Pelvis 33, no. 1 (2021): 11–17, 10.5371/hp.2021.33.1.11. PMC7952269 33748021

[95] J. C. Patzkowski and M. S. Patzkowski , “AAOS/METRC Clinical Practice Guideline Summary: Pharmacologic, Physical, and Cognitive Pain Alleviation for Musculoskeletal Extremity/Pelvis Surgery,” Journal of the American Academy of Orthopaedic Surgeons 30, no. 18 (2022): e1152–e1160, 10.5435/JAAOS-D-22-00047. 36166386

[96] Investigators NOP , A. Gazendam , S. Ekhtiari , et al., “Effect of a Postoperative Multimodal Opioid‐Sparing Protocol vs Standard Opioid Prescribing on Postoperative Opioid Consumption After Knee or Shoulder Arthroscopy: A Randomized Clinical Trial,” Journal of the American Medical Association 328, no. 13 (2022): 1326–1335, 10.1001/jama.2022.16844. PMC9533185 36194219

[97] S. T. Skou , E. M. Roos , M. B. Laursen , et al., “A Randomized, Controlled Trial of Total Knee Replacement,” New England Journal of Medicine 373, no. 17 (2015): 1597–1606, 10.1056/NEJMoa1505467. 26488691

[98] R. Tazreean , G. Nelson , and R. Twomey , “Early Mobilization in Enhanced Recovery After Surgery Pathways: Current Evidence and Recent Advancements,” Journal of Comparative Effectiveness Research 11, no. 2 (2022): 121–129, 10.2217/cer-2021-0258. 35045757

[99] J. Manrique , G. A. Komnos , T. L. Tan , S. Sedgh , N. Shohat , and J. Parvizi , “Outcomes of Superficial and Deep Irrigation and Debridement in Total Hip and Knee Arthroplasty,” Journal of Arthroplasty 34, no. 7 (2019): 1452–1457, 10.1016/j.arth.2019.03.032. 30962091

[100] H. W. Meislin , S. A. Lerner , M. H. Graves , et al., “Cutaneous Abscesses. Anaerobic and Aerobic Bacteriology and Outpatient Management,” Annals of Internal Medicine 87, no. 2 (1977): 145–149, 10.7326/0003-4819-87-2-145. 196534

[101] J. Macfie and J. Harvey , “The Treatment of Acute Superficial Abscesses: A Prospective Clinical Trial,” British Journal of Surgery 64, no. 4 (1977): 264–266, 10.1002/bjs.1800640410. 322789

[102] J. L. Del Pozo and R. Patel , “Clinical Practice. Infection Associated With Prosthetic Joints,” New England Journal of Medicine 361, no. 8 (2009): 787–794, 10.1056/NEJMcp0905029. PMC2850113 19692690

[103] A. Casenaz , L. Piroth , L. Labattut , et al., “Epidemiology and Antibiotic Resistance of Prosthetic Joint Infections According to Time of Occurrence, a 10‐Year Study,” Journal of Infection 85, no. 5 (2022): 492–498, 10.1016/j.jinf.2022.07.009. 35933039

[104] R. Chanderraj , A. J. Admon , Y. He , et al., “Mortality of Patients With Sepsis Administered Piperacillin‐Tazobactam vs Cefepime,” JAMA Internal Medicine 184, no. 7 (2024): 769–777, 10.1001/jamainternmed.2024.0581. PMC11091820 38739397

[105] M. A. Ahmad , S. Ab Rahman , and M. A. Islam , “Prevalence and Risk of Infection in Patients With Diabetes Following Primary Total Knee Arthroplasty: A Global Systematic Review and Meta‐Analysis of 120,754 Knees,” Journal of Clinical Medicine 11, no. 13 (2022): 3752, 10.3390/jcm11133752. PMC9267175 35807033

[106] A. Castro‐Balado , I. Varela‐Rey , B. Mejuto , et al., “Updated Antimicrobial Dosing Recommendations for Obese Patients,” Antimicrobial Agents and Chemotherapy 68, no. 5 (2024): e0171923, 10.1128/aac.01719-23. PMC11064535 38526051

[107] S. Deinhardt‐Emmer , B. G. Chousterman , J. C. Schefold , et al., “Sepsis in Patients Who Are Immunocompromised: Diagnostic Challenges and Future Therapies,” Lancet Respiratory Medicine 13, no. 7 (2025): 623–637, 10.1016/S2213-2600(25)00124-9. 40409328

[108] D. W. Lee , H. S. Han , and D. H. Ro , “Risk Factors of Recurrent Periprosthetic Joint Infection of the Knee After Two‐Stage Reimplantation,” Knee Surgery & Related Research 37, no. 1 (2025): 4, 10.1186/s43019-025-00258-5. PMC11731532 39810251

[109] H. Rajgor , H. Dong , R. Nandra , M. Parry , J. Stevenson , and L. Jeys , “Repeat Revision TKR for Failed Management of Peri‐Prosthetic Infection Has Long‐Term Success but Often Require Multiple Operations: A Case Control Study,” Archives of Orthopaedic and Trauma Surgery 143, no. 2 (2023): 987–994, 10.1007/s00402-022-04594-z. 35980459

