# Supplementary material for: Differentiating Normal Healing From Infection in Total Joint Arthroplasty: An Educational and Diagnostic Tool for Emergency Providers Evaluating the Postoperative Patient
Source: Acad Emerg Med. 2026 Sep 27;33(9):e70414. doi: 10.1111/acem.70414 (PMC13617165; doi:10.1111/acem.70414)
Supplement: Supplementary file 1 [file ACEM-33-0-s001.docx]

**Physiology of inflammation and normal healing**

Wound healing occurs in 4 overlapping stages: hemostasis, inflammation, proliferation, and maturation ^1^. Hemostasis begins immediately, as blood vessels vasoconstrict in response to injury ^2^. Platelets adhere to vascular walls via GPIb reception to von Willebrand factor after damage exposes von Willebrand factor, collagen, and other extracellular matrix proteins. Platelet activation is triggered by GPVI to collagen, releasing secondary mediators, such as ADP and Thromboxane A2 ^3^. These secondary mediators cause nearby platelets to aggregate, helping to produce a platelet thrombus ^4^. Cytokines and growth factors, such as platelet-derived growth factor, are also released during hemostasis and are important in later stages of wound healing ^5^. Simultaneously, the coagulation cascade begins as Tissue Factor is released from damaged endothelium, activating the extrinsic pathway, and as exposed collagen or exposed anionic surfaces activates the intrinsic cascade ^6^. Coagulation cascade creates a fibrin clot at the site of injury ^2^.

Inflammation begins during the first few days of injury, and has two important components: wound immunity and wound healing ^7^. Wound immunity focuses on fighting infection and removing debris ^8^. Wound healing focuses on repair and regeneration ^9^. PMNs are the earliest inflammatory cells to arrive, attracted by cytokines, growth factors, and bacterial products that also increase vascular permeability and cause vasodilation ^1,10^. PMNs utilize oxygen and NADPH to generate a superoxide anion, NADP+ and a hydrogen ion via NADPH oxidase.  The oxygen radicals are then used by PMNs in the oxidative burst to promote killing and wound immunity ^11^. Macrophages enter later and further assist in wound immunity via phagocytosis and leukocyte recruitment amongst other mechanisms ^12^.

The oxidative burst from PMNs also helps initiate wound healing. Additionally, immune cells adjust metabolically to pro-inflammatory signals, undergoing the Warburg Effect. Lactate is generated in an aerobically-mediated manner as activated neutrophils preferentially utilize aerobic glycolysis over oxidative phosphorylation as a means to create sufficient NADPH from the Pentose Phosphate Pathway ^13^. Hydrogen ions are also transferred out of the cells as a result of the oxidative burst ^14^. Both lactic acid and hydrogen ions activate fibroblasts and promote collagen production ^15^. Lactate aids in wound healing by promoting collagen synthesis in several mechanisms. First, lactate has been shown to increase the activity of prolyl hydroxylase, an important enzyme in collagen biosynthesis. Second, lactate down regulates ADP-ribosylation, which is the conversion of NAD+ to ADPR via ADP Ribosyl Transferase, relieving suppression on collagen transcription. This is achieved under higher concentrations of lactate, where lactate dehydrogenase is forced to work in a retrograde fashion. Lactate dehydrogenase converts lactate into pyruvate, simultaneously producing NADH from NAD+. The pool of NAD+ is subsequently decreased, thereby decreasing the production of ADPR. ADPR normally suppresses collagen transcription. Therefore, an increase in lactate helps to relieve suppression of collagen transcription ^16^. Additionally, lactate acts as an epigenetic modulator in wound healing. It affects gene expression of fibroblasts through histone lysine lactylation and by influencing post-translation modifications ^17^. Lactate likely also induces a biochemical “perception” of hypoxia within the wound regardless of oxygen level, which further promotes deposition of collagen, release of growth factors, and expression of heat shock proteins to aid in tissue repair ^18^. In a similar fashion to lactate, hydrogen ions can be used in the process to reduce NAD+ to NADH ^15^. This further decreases the pool of NAD+, increasing collagen production. An acidic state may also more readily allow that transpiration of oxygen during the relatively hypoxic state that the oxidative blast creates ^15^. Overall, the acidic pH generated during the oxidative burst has been found to stimulate fibroblast migration, proliferation, and collagen deposition ^19^.

There are several other mechanisms of inflammatory-mediated wound healing. First, inflammatory cytokines and growth factors, such as fibroblast growth factor, released by PMNs and macrophages directly activate fibrosis and angiogenesis to aid in healing ^20^. Second, reactive oxygen species, such as H2O2, produced as  a result of the oxidative burst have been found to act as signaling molecules. These molecules can subsequently activate fibroblasts and promote angiogenesis ^21,22^. Third, neutrophils may directly transport matrix components into wounds to assist in healing ^23^. In summary, inflammatory cytokines, ROS, an acidic pH, and lactate all generated in the inflammatory reaction also work together to orchestrate normal healing. These processes illustrate that inflammation is not only important in wound immunity, but also essential to physiologic wound healing.

Figure 3: The beginning phases of wound healing will take place with early inflammation following hemostasis. PMNs utilize oxygen and NADPH to generate a superoxide anion, NADP+ and a hydrogen ion via NADPH oxidase. Lactate is generated in an aerobically-mediated manner. Both lactic acid and hydrogen ions activate fibroblasts and promote collagen production. The patient in this image consented and all identifiers were removed.

**References**

1.	Grubbs H, Manna B. Wound Physiology. *StatPearls*. 2025.

2.	Broughton G, 2nd, Janis JE, Attinger CE. The basic science of wound healing. *Plast Reconstr Surg*. Jun 2006;117(7 Suppl):12S-34S. doi:10.1097/01.prs.0000225430.42531.c2

3.	Varga-Szabo D, Pleines I, Nieswandt B. Cell adhesion mechanisms in platelets. *Arterioscler Thromb Vasc Biol*. Mar 2008;28(3):403-12. doi:10.1161/ATVBAHA.107.150474

4.	Nieswandt B, Pleines I, Bender M. Platelet adhesion and activation mechanisms in arterial thrombosis and ischaemic stroke. *J Thromb Haemost*. Jul 2011;9 Suppl 1:92-104. doi:10.1111/j.1538-7836.2011.04361.x

5.	Janis JE, Harrison B. Wound Healing: Part I. Basic Science. *Plast Reconstr Surg*. Sep 2016;138(3 Suppl):9S-17S. doi:10.1097/PRS.0000000000002773

6.	Smith SA, Travers RJ, Morrissey JH. How it all starts: Initiation of the clotting cascade. *Crit Rev Biochem Mol Biol*. 2015;50(4):326-36. doi:10.3109/10409238.2015.1050550

7.	Langworthy MJ, Hankins K, Storey T. The Basic and Clinical Science of Wounds in the Austere Environment. *The Journal of Orthopaedic Experience & Innovation*. 9/5/2023 2023;4(2)doi:https://doi.org/10.60118/001c.75162

8.	Gao M, Guo H, Dong X, et al. Regulation of inflammation during wound healing: the function of mesenchymal stem cells and strategies for therapeutic enhancement. *Front Pharmacol*. 2024;15:1345779. doi:10.3389/fphar.2024.1345779

9.	Wang Z, Qi F, Luo H, Xu G, Wang D. Inflammatory Microenvironment of Skin Wounds. *Front Immunol*. 2022;13:789274. doi:10.3389/fimmu.2022.789274

10.	Witte MB, Barbul A. General principles of wound healing. *Surg Clin North Am*. Jun 1997;77(3):509-28. doi:10.1016/s0039-6109(05)70566-1

11.	Panday A, Sahoo MK, Osorio D, Batra S. NADPH oxidases: an overview from structure to innate immunity-associated pathologies. *Cell Mol Immunol*. Jan 2015;12(1):5-23. doi:10.1038/cmi.2014.89

12.	Koh TJ, DiPietro LA. Inflammation and wound healing: the role of the macrophage. *Expert Rev Mol Med*. Jul 11 2011;13:e23. doi:10.1017/S1462399411001943

13.	Manosalva C, Quiroga J, Hidalgo AI, et al. Role of Lactate in Inflammatory Processes: Friend or Foe. *Front Immunol*. 2021;12:808799. doi:10.3389/fimmu.2021.808799

14.	Ramsey IS, Ruchti E, Kaczmarek JS, Clapham DE. Hv1 proton channels are required for high-level NADPH oxidase-dependent superoxide production during the phagocyte respiratory burst. *Proc Natl Acad Sci U S A*. May 5 2009;106(18):7642-7. doi:10.1073/pnas.0902761106

15.	Langworthy MJ. Articular Reconstruction of the Elbow After High Kinetic Injury Trauma. *Techniques in Orthopaedics*. 2010;25(4):189-196. doi:10.1097/BTO.0b013e3182010640

16.	Ghani QP, Wagner S, Hussain MZ. Role of ADP-ribosylation in wound repair. The contributions of Thomas K. Hunt, MD. *Wound Repair Regen*. Nov-Dec 2003;11(6):439-44. doi:10.1046/j.1524-475x.2003.11608.x

17.	Wu H, Liang W, Han M, et al. Mechanisms regulating wound healing: Functional changes in biology mediated by lactate and histone lactylation. *J Cell Physiol*. Oct 2023;238(10):2243-2252. doi:10.1002/jcp.31122

18.	Trabold O, Wagner S, Wicke C, et al. Lactate and oxygen constitute a fundamental regulatory mechanism in wound healing. *Wound Repair Regen*. Nov-Dec 2003;11(6):504-9. doi:10.1046/j.1524-475x.2003.11621.x

19.	Sim P, Strudwick XL, Song Y, Cowin AJ, Garg S. Influence of Acidic pH on Wound Healing In Vivo: A Novel Perspective for Wound Treatment. *Int J Mol Sci*. Nov 7 2022;23(21)doi:10.3390/ijms232113655

20.	Pena OA, Martin P. Cellular and molecular mechanisms of skin wound healing. *Nat Rev Mol Cell Biol*. Aug 2024;25(8):599-616. doi:10.1038/s41580-024-00715-1

21.	Lopes FB, Sarandy MM, Novaes RD, Valacchi G, Goncalves RV. OxInflammatory Responses in the Wound Healing Process: A Systematic Review. *Antioxidants (Basel)*. Jul 9 2024;13(7)doi:10.3390/antiox13070823

22.	Ukaegbu K, Allen E, Svoboda KKH. Reactive Oxygen Species and Antioxidants in Wound Healing: Mechanisms and Therapeutic Potential. *Int Wound J*. May 2025;22(5):e70330. doi:10.1111/iwj.70330

23.	Fischer A, Wannemacher J, Christ S, et al. Neutrophils direct preexisting matrix to initiate repair in damaged tissues. *Nat Immunol*. Apr 2022;23(4):518-531. doi:10.1038/s41590-022-01166-6
