# Supplementary material for: Differentiating Normal Healing From Infection in Total Joint Arthroplasty: An Educational and Diagnostic Tool for Emergency Providers Evaluating the Postoperative Patient
Source: Acad Emerg Med. 2026 Sep 27;33(9):e70414. doi: 10.1111/acem.70414 (PMC13617165; doi:10.1111/acem.70414)
Supplement: Supplementary file 2 [file ACEM-33-0-s002.docx]

**2018 Musculoskeletal Infection Society (MSIS)/International Consensus Meeting (ISM)/Parvizi Citeria:**

- Major Criteria (PJI diagnosis if ≥ 1):
  - Same organism isolated on two separate cultures
  - Sinus tract present
- Minor Criteria (PJI diagnosis if score ≥ 6, inconclusive if score 2-5, excluded if score 0-1)
  - Serum CRP >1 mg/dL or D-dimer > 860 ng/mL, 2 points
  - Serum ESR >30 mm/hr, 1 point
  - Synovial WBC >3000 cells/μL or elevated leukocyte esterase, 3 points
  - Positive synovial alpha-defensin, 3 points
  - Synovial PMN% >80%, 2 points
  - Synovial CRP >6.9 mg/L, 1 point

**2021** **European Bone and Joint Infection Society (EBJIS) criteria:**

- Infection Confirmed (PJI diagnosis if ≥ 1):
  - Sinus tract
  - Synovial WBC >3,000 cells/μL
  - Synovial PMN% >80%
  - Positive synovial alpha defensin
  - Same organism isolated on two separate cultures
  - >50 colony forming units (CFU)/mL of any organism on synovial sonification
  - Synovial presence of ≥ five neutrophils in ≥ five high power fields
  - Presence of visible microorganisms in synovial fluid
- Infection Likely (only if positive clinical feature or elevated serum CRP with another laboratory, histological, or microbiological finding):
  - Radiologic signs of TJA loosening within 5 years of implantation
  - Previous wound healing problems
  - History of recent fever or bacteremia
  - Purulence around prosthesis site
  - CRP >1mg/dL
  - Synovial WBC >1,500 cells/μL
  - Synovial PMN% >65%
  - Joint aspirate culture positive
  - Single intraoperative tissue culture positive
  - >1 CFU/mL of any organism on synovial sonification
  - Synovial presence of ≥ five neutrophils in a single high power field
  - Positive WBC scintigraphy
- Infection unlikely (if all criteria are met):
  - Clear alternate reason for implant dysfunction
  - Synovial WBC ≤ 1,500 cells/μL
  - Synovial PMN% ≤ 65%
  - All cultures negative
  - No growth on synovial sonification
  - Negative synovial high power field

Negative three-phase isotope bone scan
